# Risk factors for in-hospital complications in patients with acute ischemic stroke: Retrospective cohort in a national reference hospital in Peru

**DOI:** 10.1016/j.heliyon.2023.e15810

**Published:** 2023-04-26

**Authors:** Miguel A. Vences, Virgilio E. Failoc-Rojas, Diego Urrunaga-Pastor, Yamilée Hurtado-Roca

**Affiliations:** a Universidad Científica del Sur, Lima, Peru; b Departamento de Neurología, Hospital Nacional Edgardo Rebagliati Martins, EsSalud, Lima, Peru; c Universidad Privada Norbert Wiener, Centro de Investigación en Medicina Traslacional, Lima, Peru; d Unidad para la Generación y Síntesis de Evidencias en Salud, Universidad San Ignacio de Loyola (USIL), Lima, Peru

**Keywords:** Stroke, Acute, Ischemic stroke, Complications, Risk factors (Source: MeSH)

## Abstract

**Objective:**

To describe the clinical and demographic characteristics of patients with acute cerebral infarction treated at a national reference hospital in Peru and determine the risk factors for in-hospital complications.

**Methods:**

We conducted a retrospective cohort study including 192 patients with acute ischemic stroke in a national reference hospital in Peru from January to September 2021. Clinical, demographic and paraclinical information was recorded from medical records. We estimated risk ratios and 95% confidence intervals using regression models with Poisson family and robust variance for the bivariate and multivariate model, adjusting for age, sex and risk factors for stroke.

**Results:**

At least one in-hospital complication occurred in 32.3% of the patients. The most frequent complications were infectious in 22.4%, followed by 17.7% of neurological complications, with other complications, such as thromboembolism, immobility and miscellaneous, being much less frequent. Regression analysis showed that stroke severity (RR = 1.76; 95%CI:1.09–2.86) and albumin greater than 3.5 mg/dL (RR = 0.53; 95%CI: 0.36–0.79) were independent risk factors for the presence of in-hospital complications.

**Conclusions:**

A high rate of in-hospital complications were observed, among which infectious and neurological complications were the most frequent. Stroke severity was a risk factor and albumin greater than 3.5 mg/dL was a protective factor for the incidence of in-hospital complications. These results can serve as a starting point for establishing stroke care systems that consider differentiated flows for the prevention of in-hospital complications.

## 1 Introduction

Stroke is one of the leading causes of disability and mortality worldwide [1]. This condition more seriously affects low- and middle-income countries, generating, according to international reports, a high health cost, not only due to hospital expenses but also due to the indirect expenses that it entails in the long term, such as those related to the loss of work of the patient [[2], [3], [4]].

Previous studies in Europe and Asia have described incidences of in-hospital complications greater than 40% in patients admitted with stroke [5,6]. In addition, it has been reported that part of the disability and mortality associated with stroke is sustained by the presence of in-hospital complications, many of which could be preventable [6]. Therefore, some measures have been proposed, such as the optimization of comprehensive care protocols and training related to preventive care and timely management of complications for all health personnel involved in the care of patients with stroke [7].

In Latin America, where most countries are low- and middle-income, there may be differences in the rate of in-hospital complications compared to what is reported in other regions. Few studies are available and most provide partial data, reporting only non-neurological complications and their predictive factors [8,9]. The main objective of this study was to determine the risk factors for in-hospital complications in patients with acute ischemic stroke treated at a national reference hospital in Peru. This article was written following the STROBE guidelines for observational studies [10].

## 2 Methods

### 2.1 Design and population

We conducted a retrospective study of a cohort of patients treated at an essential stroke center belonging to the social security system in Lima, Peru. The Edgardo Rebagliati Martins National Hospital is a national reference hospital with resolution capacity for intravenous reperfusion treatment.

The population included all patients over 18 years of age admitted to the emergency department (ED) with a diagnosis of acute ischemic stroke during the period from January 1 to September 30, 2021. The information of the patients enrolled was obtained from the data recorded in the clinical history during hospitalization (from admission to the ED until discharge or death). A diagnosis of acute ischemic stroke was given to patients with acute neurological deficit confirmed by neuroimaging (tomography or cerebral magnetic resonance) and with a maximum stroke-gate time of 72 h. A total of 215 patients attended the ED with this diagnosis during the study period; of these, we excluded 23 patients diagnosed with COVID-19 at admission or during hospitalization, leaving 192 patients for data analysis.

### 2.2 Variables

In-hospital complications were defined as medical problems arising during hospital stay according to clinical definitions [5,6]. We made an adaptation of the previously used classification, and the complications were classified as neurological (seizures, recurrent stroke, hemorrhagic transformation, intracranial hypertension and unexplained events), infectious (thoracic, urinary or other infection), thromboembolism (pulmonary and deep vein thrombosis), immobility complications (pressure ulcers and falls) and miscellaneous (not classified in the previous categories). Due to the retrospective design, we did not include pain or psychological complications, because they were not reported in the medical record.

We evaluated demographic variables such as age, sex, level of education, toxic habits (alcohol, smoking and drug use), previous medication for cardiovascular disease as well as the most prevalent pathological antecedents including high blood pressure, diabetes mellitus, dyslipidemia, obesity, chronic kidney disease, chronic heart disease, cardiac arrhythmia, autoimmune disease, chronic lung disease, malignant neoplasm, cerebrovascular disease, thyroid disease, obstructive sleep area syndrome, and chronic liver disease. Based on previous comorbidities, the age adjusted Charlson comorbidity index (CCI) [11] was also used.

Regarding clinical variables, the following were determined: baseline status (estimated by the “modified Rankin scale”, mRS at admission) [12], vital functions at admission (heart rate, systolic and diastolic blood pressure) and the clinical picture presented in the patient. We recorded the severity of acute ischemic stroke at ED admission and at discharge estimated by the “National Institutes of Health Stroke Scale” (NIHSS) and divided into groups: mild (0–4), moderate (5–15), severe (16–25) and very severe (>25) [13]. We used the etiological classification of the “Trial of ORG 10172 in Acute Stroke Treatment” (TOAST) for cerebral infarction [14].

The following laboratory tests were performed at admission (carried out in the first 72 h of admission): capillary glycemia test, hemoglobin (Hb), leukocytes, neutrophil/lymphocyte ratio (NLR), lactate dehydrogenase (LDH), total protein, albumin, C-reactive protein (CRP) and D-dimers. Imaging studies were performed to determine the presence of previous vascular lesions, cerebral vascular territory (supratentorial and infratentorial), laterality, leukoaraiosis, microbleeding, early signs of stroke and hemorrhagic transformation. Treatment variables included intravenous reperfusion, surgical treatment, and the type of secondary prevention treatment (single/dual antiplatelet therapy or anticoagulation).

During follow-up, admission to the intermediate care unit, intensive care unit (ICU), functional outcome (good, mRS 0–2 and poor, mRS 3–6) and the destination of the patient (discharge or death) were recorded.

### 2.3 Statistical analysis

The information was recorded and stored anonymously in a database to protect confidentiality and was only accessible by the principal investigator. Subsequently, the database was exported to the STATA v17 program. To summarize the categorical variables, frequencies and percentages were used, while for the quantitative variables, measures of central tendency and dispersion were used depending on the distribution of the data.

To evaluate the associations between in-hospital complications and sociodemographic, clinical, and paraclinical variables, hypothesis tests were performed according to the nature of the independent variables. The Chi-square test was used for categorical variables, while the Student’s *t*-test or Mann-Whitney *U* test was used for quantitative variables.

Finally, the effect size (relative risk and 95% confidence intervals) was estimated by creating Poisson regression models with robust variance. Crude models were constructed in the bivariate analysis considering the following factors: age, sex, history of stroke, baseline status (independent, mRS 0–2 and dependent, mRS 3–5), use of previous medication for cardiovascular disease, presence of previous comorbidity, CCI (low, 0–4 and high, greater than 4), vital functions on admission, clinical picture, severity of stroke (non-severe, 0–10 and severe, greater than 10), length of stay in the ED and laboratory tests (Hb, leukocytes, NLR, LDH, total protein, albumin, CRP, and D-dimer).

The multivariate analysis was adjusted for age, sex and risk factors for stroke (hypertension, diabetes, history of stroke). In addition, a parsimonious model was constructed, performing a nesting model among the variables that were statistically significant in the crude analysis model. The assumption of linearity was evaluated when constructing the models and, additionally, lost data among the variables and collinearity were evaluated in the adjusted models. A statistical significance level of 0.05 was used for all the statistical tests.

### 2.4 Ethical issues

In the present study, only the researchers had access and reviewed the clinical histories, then, there was no direct contact with the patients. The study was carried out in accordance with the Declaration of Helsinki, and the authors ensured anonymity of the patient data included in the study. In addition, the research protocol was approved by the Ethics Committee of the hospital (Letter No. 964 -GRPR-ESSALUD-2021) and the Ethics Committee of the Universidad Científica del Sur (Letter No. 404-CIEI-CIENTÍFICA-2021).

## 3 Results

### 3.1 Demographic characteristics of the participants

During the study period, we included 192 patients diagnosed with acute ischemic stroke. The mean age was 71.7 ± 11.9 years, 16.2% of the patients were under 60 years of age, and 64.6% were male. In addition, 74.3% of the patients presented at least one previous comorbidity in the clinical history, and the most frequent was arterial hypertension (67.2%) and diabetes mellitus (30.7%). We found that 36.1% of the patients reported no prior medication for cardiovascular disease, while 57.3% used antihypertensive drugs and 21.4% antiplatelet therapy. Further details of the demographic characteristics of the patients are shown in Table 1.

**Table 1:** Demographic characteristics of patients with acute ischemic stroke (n = 192).

Variables	n	%
**Sex**		
Female	68	35.4
Male	124	64.6
**Age (years)**		
30 to 59	31	16.2
60 to 70	50	26
≥71	111	57.8
**Baseline status**		
Independent (mRS:0–2)	183	95.3
Dependent (mRS:3–5)	9	4.7
**Comorbidities**		
None	50	25.7
At least 1	142	74.3
Hypertension	129	67.2
Diabetes mellitus	59	30.7
Chronic kidney disease	12	6.3
Chronic coronary disease	14	7.3
Epilepsy	10	5.2
**History of previous stroke**		
None	154	80.2
Hemorrhagic stroke	2	1
Ischemic stroke	35	18.2
**CCI**		
0	9	4.7
1 to 2	26	13.5
3 to 4	72	37.5
5 or more	85	44.3
**Previous medication**		
No	69	36.1
Yes	122	63.9
Antihypertensive	110	57.3
Antipletelet therapy	41	21.4
Statins	30	15.6
Antiarrhythmics	34	17.7
Warfarin	10	5.2

*Abbreviations:* mRS: Modified Rankin Scale; CCI: age adjusted Charlson comorbidity index.

### 3.2 Clinical characteristics of the study sample

Among the admitted patients, 95.3% had a baseline status of functional independence. The most frequent initial symptoms at admission were limb weakness (75.8%) and dysarthria (49.2%). Disease severity was mild in 26% of the patients, being moderate in 54.2%, severe in 18.2% and very severe in 1.6%. Regarding the etiological classification, 39.1% were reported as long-artery atherosclerosis, 25% cardioembolic, 22.9% lacunar, 2.1% had infrequent causes, and 10.9% had an undetermined etiology. Neuroimaging findings showed that 83.8% corresponded to the supratentorial territory, while the middle cerebral artery was the most reported vascular territory and 31.2% had some degree of leukoaraiosis.

With respect to treatment, of the 24 patients (12.5%) admitted within the therapeutic window (stroke-door time of up to 4.5 h), only 5 patients (2.6%) received acute treatment with recombinant tissue plasminogen activator (rtPA) as prevention strategies. Secondary antiplatelet therapy or anticoagulation was used according to the etiological classification.

In relation to the clinical evolution of the patients, 60 (31.2%) were admitted to an intermediate care unit and 6 (3.1%) to an ICU. The median stay in the ED before admission to a neurological unit was 2 days (interquartile range [IQR], 1–3 days) and the median hospital stay was 15 days (IQR, 10–23 days). As a clinical outcome, a mortality of 6.2% was reported. Regarding functional results, 49.5% of the total number of patients admitted to the study were functionally dependent; the 5 patients who received rtPA were functionally independent. Table 2 shows the clinical and imaging characteristics of the patients studied.

**Table 2:** Clinical and imaging characteristics of patients with acute ischemic stroke (n = 192).

Variables	n	%
**Stroke severity (NIHSS admission)**		
I (0–4)	50	26
II (5–15)	104	54.2
III (16–25)	35	18.2
IV (26 or more)	3	1.6
**Stroke symptom**		
Limb weakness	144	75.8
Dysarthria	94	49.2
Headache	39	20.4
Aphasia	30	15.7
Ataxia	28	14.7
**Etiological classification**		
Large vessel atherothrombotic	75	39.1
Lacunar	44	22.9
Cardioembolic	48	25
Uncommon causes	4	2.1
Undetermined etiology	21	10.9
**Neuroimaging findings**		
**Cerebral vascular territory**		
Infratentorial	31	16.2
Supratentorial	161	83.8
**Affected cerebral artery**		
MCA	121	63
ACA	3	1.6
PCA	10	5.2
VB	29	15.1
2 or more territories	29	15.1
**Imaging laterality**		
Left	104	54.2
Right	88	45.8
**Leukoaraiosis**	59	31.2
**Microbleeding**	7	3.7
**Early signs of stroke**	8	4.2
**Treatment received**		
Thrombolysis	5	2.6
Simple antiplatelet therapy	111	57.8
Dual antiplatelet therapy	7	3.6
Anticoagulation	43	22.4
Statins	141	73.8
**Intermediate care unit admission**	60	31.2
**ICU admission**	6	3.1
**In-hospital complication**	62	32.3
**NIHSS at discharge**		
I (0–4)	52	28.9
II (5–15)	101	56.1
III (16–25)	26	14.4
IV (26 or more)	1	0.6
**NIHSS discharge in patients with thrombolysis**		
I (0–4)	1	20
II (5–15)	4	80
**Rankin at discharge**		
Independent (mRS:0–2)	85	44.3
Dependent	95	49.5
(mRS:3–5)		
Death	12	6.2

*Abbreviations:* MCA: middle cerebral artery, ACA: anterior cerebral artery, PCA: posterior cerebral artery, VB: vertebrobasilar territory; ICU: Intensive Care Unit; mRS: Modified Rankin Scale.

### 3.3 In-hospital complications

During hospitalization, at least one in-hospital complication occurred in 32.3% of patients. Regarding the types of complications, the most frequent were infectious in 22.4% (thoracic 15.1% and urinary 7.3%), followed by 17.7% of neurological complications (recurrent stroke 1.6%, epileptic seizures 1.6%, 5.3% hemorrhagic transformation, intracranial hypertension 3.6% and unexplained events 7.3%), while other complications, such as thromboembolism (deep vein thrombosis 0.5%), immobility (pressure ulcers 5.2%) and miscellaneous (minor bleeding 1%, major bleeding 1.6%, acute renal failure 7.3%, hydroelectrolytic disorder 2.6%) were much less frequent. The coexistence of the 2 most frequent types of complications (neurological and infectious) was reported in 12 patients (6.25%).

When comparing the clinical, demographic and laboratory characteristics of the patients with acute ischemic stroke presenting in-hospital complications with the group without complications, there were significant differences in age (age difference between complicated versus uncomplicated was 4.52 years; p = 0.013), the presence of at least one comorbidity (34.9% comorbility and complication versus 13.0% in not comorbility and complication; p = 0.035), high CCI (5 or more have 42.3% with complication; p = 0.008), previous use of antiarrhythmics (p = 0.001), severity of stroke according to an NIHSS >10 (p = 0.002), etiological classification of stroke (p = 0.006) and in all laboratory tests at admission (Hb, leukocytes, NLR, LDH, total protein, albumin, CRP and D-dimer).

Significant differences were found in the following variables in the exploratory analysis by type of complication in patients with neurological complications: systolic blood pressure at admission (lower systolic blood pressure presented a higher percentage of neurological complications compared to higher 185 mmgHg 20.5 vs. 4.0; p = 0.047), previous use of antiarrhythmics (p = 0.001), longer ED stay (p = 0.01), stroke severity according to the NIHSS > 10 (p = 0.007), etiological classification of stroke (p = 0.005), headache (p = 0.004), dysarthria (p = 0.011) and only Hb, leukocyte and CRP values were significant. Regarding infectious complications, significant differences were found in patients with a history of previous medication (p = 0.010), use of antihypertensive drugs (p = 0.04), and hypoalbuminemia (p = 0.043) (Table 3, Table 4, Table 5).

**Table 3:** Comparison of in-hospital complications and type of complication according to the demographic characteristics of patients with acute ischemic stroke.

Variables	n-hospital complication			Neurological complication			Infectious complication		
	Yes	No	P value	Yes	No	P value	Yes	No	P value
	n = 62 (%)	n (%)		n = 34 (%)	n (%)		n = 42 (%)	n (%)	
**Age**	74.74 (10.7)	70.22 (12.19)	0.013	73.5 (14.4)	71.3 (11.3)	0.407	73.1 (11.3)	71.2 (12.1)	0.373
**Sex**									
Female	18 (26.5)	50 (73.5)	0.201	14 (20.6)	54 (79.4)	0.439	15 (22.4)	52 (77.6)	0.922
Male	44 (35.5)	80 (64.5)		20 (16.1)	104 (83.9)		27 (21.8)	97 (78.2)	
**Stroke history**									
No	52 (33.5)	103 (66.5)	0.446	28 (18.1)	127 (81.9)	0.791	36 (23.4)	118 (76.6)	0.345
Yes	10 (27.0)	27 (73.0)		6 (16.2)	31 (83.8)		6 (16.2)	31 (83.8)	
**Comorbidities**									
No	3 (13.0)	20 (90.0)	0.035	3 (13.0)	20 (87.0)	0.532	3 (13.0)	20 (87.0)	0.269
Yes	59 (34.9)	110 (65.1)		31 (18.3)	138 (81.7)		39 (23.2)	129 (76.8)	
**CCI**									
0 to 4	26 (24.3)	81 (75.5)	0.008	15 (14.0)	92 (86.0)	0.133	19 (17.8)	88 (82.2)	0.111
5 or more	36 (42.3)	49 (57.7)		19 (22.4)	66 (77.6)		23 (27.4)	61 (72.6)	
**Previous medication**									
No	21 (30.4)	48 (69.6)	0.653	15 (21.7)	54 (78.3)	0.285	8 (11.8)	60 (88.2)	0.010
Yes	41 (33.6)	81 (66.4)		19 (15.8)	103 (84.4)		34 (27.9)	88 (72.1)	
**Antiplatelet therapy**									
No	48 (31.8)	103 (68.2)	0.775	27 (17.9)	124 (82.1)	0.904	30 (20.0)	120 (80.0)	0.204
Yes	14 (34.2)	27 (65.8)		7 (17.0)	34 (83.0)		12 (29.3)	29 (70.7)	
**Warfarin**									
No	57 (31.3)	125 (68.7)	0.219	33 (18.1)	149 (81.9)	0.512	40 (22.1)	141 (77.9)	0.876
Yes	5 (50.0)	5 (50.0)		1 (10.0)	9 (90.0)		2 (20.0)	8 (80.0)	
**Statins**									
No	52 (32.1)	110 (67.9)	0.894	28 (17.3)	134 (82.7)	0.72	33 (20.5)	128 (79.5)	0.249
Yes	10 (33.3)	20 (66.7)		6 (20.0)	24 (80.0)		9 (30.0)	21 (70.0)	
**NOAC**									
No	61 (32.5)	127 (67.5)	0.753	32 (17.0)	156 (83.0)	0.087	41 (21.9)	146 (78.1)	0.883
Yes	1 (25.0)	3 (75.0)		2 (50.0)	2 (50.0)		1 (25.0)	3 (75.0)	
**Antihypertensive**									
No	28 (34.2)	54 (65.8)	0.635	18 (21.9)	64 (78.1)	0.184	12 (14.8)	69 (85.2)	0.040
Yes	34 (30.9)	76 (69.1)		16 (14.5)	94 (84.5)		30 (27.3)	80 (72.7)	
**Anti-arrhythmic**									
No	43 (27.2)	115 (72.8)	0.001	21 (13.3)	137 (86.7)	0.001	32 (20.4)	125 (79.6)	0.249
Yes	19 (55.9)	15 (44.1)		13 (38.2)	21 (61.8)		10 (29.4)	24 (70.6)	
**Immunosuppressants**									
No	60 (32.3)	126 (67.7)	0.956	32 (17.2)	154 (82.8)	0.308	40 (21.6)	145 (78.4)	0.495
Yes	2 (33.3)	4 (66.7)		2 (33.3)	4 (66.7)		2 (33.3)	4 (66.7)	
Emergency stay*‡	2 (1–3)	2 (1–2)	0.105	2 (1–2)	2 (1–2)	0.010	2 (1–3)	2 (1–2)	0.051

*Abbreviations:* CCI: age adjusted Charlson comorbidity index; NOAC: Novel oral anticoagulants.

*Median (IQR: interquartile range).

We used the following statistical tests: chi2 test and ‡ Mann-Whitney *U* test.

**Table 4:** Comparison of in-hospital complications and type of complication according to the clinical characteristics and imaging findings of patients with acute ischemic stroke.

Variables	In-hospital complication			Neurological complication			Infectious complication		
	Yes	No	P value	Yes	No	P value	Yes	No	P value
	n = 62 (%)	n (%)		n = 34 (%)	n (%)		n = 42 (%)	n (%)	
**SBP (mmHg)**									
≤185	50 (31.1)	111 (68.9)	0.095	33 (20.5)	128 (79.5)	0.047	35 (21.9)	125 (78.1)	0.497
>185	12 (48.0)	13 (52.0)		1 (4.0)	24 (96.0)		7 (28.0)	18 (72.0)	
**DBP (mmHg)**									
≤110	62 (33.5)	123 (66.5)	0.478	34 (18.4)	151 (81.6)	0.635	41 (22.3)	143 (77.7)	0.064
>110	0 (0.0)	1 (100)		0 (0.0)	1 (100.0)		1 (100.0)	0 (0.0)	
**HR (beats/min)**									
≤90	44 (31.2)	97 (68.8)	0.068	25 (17.7)	116 (82.3)	0.832	34 (24.1)	107 (75.9)	0.197
>90	15 (48.4)	16 (51.6)		5 (16.1)	26 (83.9)		4 (13.3)	26 (86.7)	
**CGT (mg/dL)**									
≤180	22 (33.9)	43 (66.1)	0.167	11 (16.9)	54 (83.1)	0.658	12 (18.5)	53 (81.5)	0.298
>180	1 (11.1)	8 (88.9)		1 (11.1)	8 (88.9)		3 (33.3)	6 (66.7)	
**Severity of stroke (NIHSS)**									
≤10	25 (23.2)	83 (76.8)	0.002	12 (11.1)	96 (88.9)	0.007	25 (23.2)	83 (76.8)	0.659
>10	37 (44.1)	47 (55.9)		22 (26.2)	62 (73.8)		17 (20.5)	66 (79.5)	
**Baseline status**									
Independent	60 (32.8)	123 (67.2)	0.508	33 (18.0)	150 (82.0)	0.595	40 (22.0)	142 (78.0)	0.986
Dependent	2 (22.2)	7 (77.8)		1 (11.1)	8 (88.9)		2 (22.2)	7 (77.8)	
**Etiological classification**									
Large vessel atherothrombotic	23 (30.7)	52 (69.3)	0.006	11 (14.7)	64 (85.3)	0.005	14 (18.7)	61 (81.3)	0.584
Lacuna	7 (15.9)	37 (84.1)		4 (9.1)	40 (90.9)		12 (27.9)	31 (72.1)	
Cardioembolic	23 (47.9)	25 (52.1)		17 (35.4)	31 (64.6)		12 (25.0)	36 (75.0)	
Uncommon causes	3 (75.0)	1 (25.0)		0 (0.0)	4 (100.0)		0 (0.0)	4 (100.0)	
Undetermined etiology	6 (28.6)	15 (71.4)		2 (9.5)	19 (90.5)		4 (19.1)	17 (80.9)	
**Limb weakness**									
No	15 (32.6)	31 (67.4)	0.997	11 (23.9)	35 (76.1)	0.221	7 (15.2)	39 (84.8)	0.189
Yes	47 (32.6)	97 (67.4)		23 (16.0)	121 (84.0)		35 (24.5)	108 (75.5)	
**Visual deficit**									
No	58 (32.9)	118 (67.1)	0.736	31 (17.6)	145 (82.4)	0.720	41 (23.4)	134 (76.6)	0.158
Yes	4 (28.6)	10 (71.4)		3 (21.4)	11 (78.6)		1 (7.1)	13 (92.9)	
**Aphasia**									
No	50 (31.1)	111 (68.9)	0.337	28 (17.4)	133 (82.6)	0.732	37 (23.0)	124 (77.0)	0.493
Yes	12 (40.0)	18 (60.0)		6 (20.0)	24 (80.0)		5 (17.2)	24 (84.8)	
**Headache**									
No	52 (34.2)	100 (65.8)	0.308	21 (13.8)	131 (86.2)	0.004	33 (21.9)	118 (78.1)	0.870
Yes	10 (25.6)	29 (74.4)		13 (33.3)	26 (66.7)		9 (23.1)	30 (76.9)	
**Epileptic seizures**									
No	59 (32.1)	125 (67.9)	0.748	33 (17.9)	151 (82.1)	0.693	42 (23.0)	141 (77.0)	0.125
Yes	3 (37.5)	5 (62.5)		1 (12.5)	7 (87.5)		0 (0.0)	8 (100.0)	
**Vertigo**									
No	58 (32.9)	118 (67.1)	0.618	31 (17.6)	145 (82.4)	0.817	40 (22.9)	135 (77.1)	0.394
Yes	4 (26.7)	11 (73.3)		3 (20.0)	12 (80.0)		2 (13.3)	13 (86.7)	
**Facial paralysis**									
No	54 (32.9)	110 (67.1)	0.735	30 (18.3)	134 (81.7)	0.662	35 (21.5)	128 (78.5)	0.605
Yes	8 (29.6)	19 (70.4)		4 (14.8)	23 (85.2)		7 (25.9)	20 (74.1)	
**Loss of sensation**									
No	61 (34.1)	118 (65.9)	0.065	34 (19.0)	145 (81.0)	0.096	42 (23.6)	136 (76.4)	0.057
Yes	1 (8.3)	11 (91.7)		0 (0.0)	12 (100.0)		0 (0.0)	12 (100.0)	
**Unsteady gait**									
No	54 (33.1)	109 (66.9)	0.634	28 (17.2)	135 (82.8)	0.587	33 (20.4)	129 (79.6)	0.166
Yes	8 (28.6)	20 (71.4)		6 (21.4)	22 (78.6)		9 (32.1)	19 (67.9)	
**Dysarthria**									
No	32 (33.0)	65 (67.0)	0.874	24 (24.7)	73 (75.3)	0.011	18 (18.6)	79 (81.4)	0.229
Yes	30 (31.9)	64 (68.1)		10 (10.6)	84 (89.4)		24 (25.8)	69 (74.2)	
**Cerebral vascular territory**									
Infratentorial	10 (32.3)	21 (67.3)	0.997	3 (9.7)	28 (90.3)	0.201	7 (23.3)	23 (76.7)	0.847
Supratentorial	52 (32.3)	109 (67.3)		31 (19.3)	130 (80.8)		35 (21.7)	126 (78.3)	
**Affected cerebral artery**									
MCA	41 (33.9)	80 (66.1)	0.892	21 (17.4)	100 (82.6)	0.075	28 (23.3)	92 (76.7)	0.781
ACA	1 (33.3)	2 (66.7)		2 (66.7)	1 (33.3)		0 (0.0)	3 (100.0)	
PCA	3 (30.0)	7 (70.0)		3 (30.0)	7 (70.0)		3 (30.0)	7 (70.0)	
VB	7 (24.1)	22 (75.9)		2 (6.9)	27 (93.1)		5 (17.2)	24 (82.8)	
2 or more territories	10 (34.5)	19 (65.5)		6 (20.7)	23 (79.3)		6 (20.7)	23 (79.3)	
**Imaging laterality**									
Left	35 (33.7)	69 (66.4)	0.661	21 (20.2)	83 (79.8)	0.327	22 (21.2)	82 (78.9)	0.76
Right	27 (30.7)	61 (69.3)		13 (14.8)	75 (85.2)		20 (23.0)	67 (77.0)	
**Leukoaraiosis**									
No	38 (29.2)	92 (70.8)	0.12	22 (16.9)	108 (83.1)	0.571	30 (23.1)	100 (76.9)	0.717
Yes	24 (40.7)	35 (59.3)		12 (20.3)	47 (79.7)		12 (20.7)	46 (79.3)	

*Abbreviations:* SBP: systolic blood pressure; DBP: SBP: diastolic blood pressure; HR: heart rate; CGT: capillary glycemia test; MCA: middle cerebral artery, ACA: anterior cerebral artery, PCA: posterior cerebral artery, VB: vertebrobasilar territory.

We used the following statistical tests: chi2 test.

**Table 5:** Comparison of in-hospital complications and type of complication according to laboratory test results of patients with acute ischemic stroke.

	In-hospital complication			Neurological complication			Infectious complication		
Variable	Yes	No	P value	Yes	No	P value	Yes	No	P value
	n = 62 (%)	n (%)		n = 34 (%)	n (%)		n = 42 (%)	n (%)	
**Hb (g/dL) †**	12.7 (1.8)	13.6 (1.9)	0.003	12.4 (1.9)	13.5 (1.8)	0.035	13.3 (2.1)	13.3 (1.8)	0.946
**Leukocytes (cells/μL)**									
<5000	7 (50.0)	7 (50.0)	0.001	4 (28.6)	10 (71.4)	0.022	2 (14.3)	12 (85.7)	0.713
5000-12000	41 (27.3)	109 (72.7)		20 (13.3)	130 (86.7)		33 (22.0)	117 (78.0)	
>12,000	11 (68.8)	5 (31.3)		6 (37.5)	10 (62.5)		4 (26.7)	11 (73.3)	
**NLR**									
<3	20 (22.7)	68 (77.3)	0.005	12 (13.6)	76 (86.4)	0.286	18 (20.5)	70 (79.5)	0.671
≥3	39 (42.4)	53 (57.6)		18 (19.6)	74 (80.4)		21 (23.1)	70 (76.9)	
**LDH (IU/L)**									
≤350	16 (25.8)	46 (74.2)	0.003	11 (17.7)	51 (82.3)	0.195	13 (21.0)	49 (79.0)	0.624
>350	12 (63.2)	7 (36.8)		6 (31.6)	13 (68.4)		5 (26.3)	14 (73.7)	
**Total proteins (g/dL)**									
≤6	15 (55.6)	12 (44.4)	0.011	6 (22.2)	21 (77.8)	0.549	8 (30.8)	18 (69.2)	0.293
>6	38 (29.9)	89 (70.1)		22 (17.3)	105 (82.7)		27 (21.3)	100 (78.7)	
**Albumin (g/dL)**									
≤3.5	14 (60.9)	9 (39.1)	0.002	6 (26.1)	17 (73.9)	0.192	9 (40.9)	13 (59.1)	0.043
>3.5	33 (27.5)	87 (72.5)		18 (15.0)	102 (85.0)		25 (20.8)	95 (79.2)	
**CRP (mg/dL)**									
≤2	20 (20.6)	77 (79.4)	<0.001	11 (11.3)	86 (88.7)	0.001	20 (20.6)	77 (79.4)	0.422
>2	29 (63.0)	17 (37.0)		16 (34.8)	30 (65.2)		12 (26.7)	33 (73.3)	
**D-dimer (μg/mL)**									
≤1	6 (20.0)	24 (80.0)	0.012	4 (13.3)	26 (86.7)	0.203	9 (30.0)	21 (70.0)	0.518
>1	21 (48.8)	22 (51.2)		11 (25.6)	32 (74.4)		10 (23.3)	33 (76.7)	

*Abbreviations:* Hb: hemoglobin; NLR: neutrophil/lymphocyte ratio; LDH: lactate dehydrogenase; CRP: C-reactive protein.

*Median (IQR: interquartile range).

We used the following statistical tests: chi2 test and † Student’s *t*-test.

### 3.4 Risk factors for in-hospital complications in the bivariate analysis

When evaluating associations with Poisson regression models, a CCI > 4 was found to increase the risk of presenting an in-hospital complication by 74% (relative risk [RR]: 1.74; 95% confidence interval [CI]: 1.15–2.65). In addition, patients with a NIHSS > 10 at admission had a 1.90-fold (95% CI: 1.25–2.9) greater risk of presenting an in-hospital complication compared to those with a lower NIHSS. All the laboratory tests presented statistically significant results in the crude analysis (Table 6).

**Table 6:** Bivariate analysis of risk factors for in-hospital complications in patients with acute ischemic stroke.

Variable	RR	95% CI	P value
**Sex**			
Female	Ref		
Male	1.34	0.84–2.13	0.215
**Age**			
≤70	Ref.	0.86–2.05	0.203
>70	1.33		
**Stroke history**			
No	Ref.		
Yes	0.81	0.45–1.43	0.462
**SBP (mmHg) (n = 186)**			
≤185	Ref.		
>185	1.55	0.97–2.47	0.069
**HR (beats/min) (n = 186)**			
≤90	Ref.		
>90	1.55	0.99–2.40	0.051
**CGT (mg/dL) (n = 74)**			
≤180	Ref		
>180	0.33	0.49–2.18	0.248
**Comorbidities**			
No	Ref		
Yes	2.68	0.91–7.87	0.073
**CCI**			
0 to 4	Ref		
5 or more	1.74	1.15–2.65	0.009
**Previous medication**			
No	Ref.		
Yes	1.1	0.71–1.71	0.656
**Stroke severity (NIHSS)**			
≤10	Ref.		
	1.9	1.25–2.90	0.003
**Baseline status**			
Independent	Ref.		
Dependent	1.02	0.81–1.29	0.841
**Imaging laterality**			
Left	Ref		
Right	0.91	0.60–1.38	0.662
**Hb (g/dL)**	0.86	0.78–0.94	0.001
**Leukocytes (cells/μL) (n = 180)**			
5000-12000	Ref.		
<5000	1.83	1.02–3.29	0.044
	2.52	1.65–3.84	<0.001
**NLR (n = 180)**			
<3	Ref		
≥3	1.87	1.18–2.94	0.007
**LDH (IU/L) (n = 81)**			
≤350	Ref.		
>350	2.45	1.42–4.23	0.001
**Total Proteins (g/dL) (n = 154)**			
≤6	Ref.		
>6	0.54	0.35–0.83	0.005
**Albumin (g/dL) (n = 143)**			
≤3.5	Ref		
>3.5	0.45	0.29–0.70	<0.001
**CRP (mg/dL) (n = 143)**			
≤2	Ref		
>2	3.06	1.95–4.80	<0.001
**D-dimer (μg/mL) (n = 73)**			
≤1	Ref		
>1	2.44	1.12–5.35	0.026

*Abbreviations:* SBP: systolic blood pressure; HR: heart rate; CGT: capillary glycemia test; CCI: age adjusted Charlson comorbidity index; Hb: hemoglobin; NLR: neutrophil/lymphocyte ratio; LDH: lactate dehydrogenase; CRP: C-reactive protein; CI: confidence interval; RR: relative risk.

An exploratory analysis was made according to the most frequent types of complications: neurological and infectious. Regarding neurological complications, patients with a NIHSS > 10 at admission had a 2.36-fold (95% CI: 1.24–4.49) greater risk of presenting a neurological complication compared to those with a lower NIHSS. Hb, leukocyte and CRP values were statistically significant. In the crude analysis of infectious complications, patients with a history of previous medication had a 2.37-fold higher risk (95% CI: 1.16–4.83) of presenting some type of infectious complication and albumin levels >3.5 mg/dL was a protective factor, decreasing the risk by 49% (95% CI: 0.28–0.94) (Table 7).

**Table 7:** Bivariate analysis of risk factors for neurological and infectious complications in patients with acute ischemic stroke.

Variable	Neurological complications			Infectious complications		
	RR	95% CI	P value	RR	95% CI	P value
**Sex**						
Female	Ref			Ref		
Male	0.78	0.42–1.45	0.438	0.97	0.56–1.67	0.922
**Age**						
≤70	Ref.			Ref.		
>70	1.18	0.63–2.22	0.61	1.33	0.75–2.33	0.327
**Stroke history**						
No	Ref.			Ref.		
Yes	0.90	0.40–2.01	0.793	0.69	0.32–1.53	0.363
**SBP (mmHg)**						
≤185	Ref.			Ref.		
>185	0.20	0.03–1.37	<0,001	1.28	0.64–2.57	0.487
**HR (beat/min)**						
≤90	Ref.			Ref.		
>90	0.91	0.38–2.19	0.833	0.55	0.21–1.45	0.227
**CGT (mg/dL)**						
≤180	Ref			Ref		
>180	0.66	0.09–4.56	0.67	1.81	0.62–5.23	0.276
**Comorbidities**						
No	Ref			Ref		
Yes	1.41	0.47–4.25	0.545	1.78	0.60–5.31	0.301
**CCI**						
0 to 4	Ref			Ref		
5 or more	1.59	0.86–2.95	0.138	1.54	0.90–2.64	0.114
**Previous medication**						
No	Ref.			Ref.		
Yes	0.72	0.39–1.32	0.285	2.37	1.16–4.83	0.018
**Stroke severity (NIHSS)**						
≤10	Ref.			Ref.		
>10	2.36	1.24–4.49	0.009	0.88	0.51–1.53	0.661
**Baseline status**						
Independent	Ref.			Ref.		
Dependent	0.61	0.09–4.03	0.613	1.01	0.29–3.55	0.986
**Imaging laterality**						
Left	Ref			Ref		
Right	0.73	0.39–1.38	0.333	1.09	0.64–1.86	0.761
**Hb (g/dL)**	0.8	0.70–0.91	0.001	0.99	0.85–1.17	0.95
**Leukocytes (cells/μL)**						
5000-12000	Ref.			Ref.		
<5000	2.15	0.85–5.41	0.107	0.65	0.17–2.43	0.522
>12,000	2.81	1.32–5.98	0.007	1.21	0.50–2.96	0.673
**NLR**						
<3	Ref			Ref		
≥3	1.43	0.73–2.81	0.292	1.13	0.65–1.97	0.672
**LDH (IU/L)**						
≤350	Ref.			Ref.		
>350	1.78	0.76–4.19	0.187	1.26	0.51–3.09	0.621
**Total proteins (g/dL)**						
≤6	Ref.			Ref.		
>6	0.78	0.35–1.74	0.544	0.69	0.35–1.35	0.279
**Albumin (g/dL)**						
≤3.5	Ref			Ref		
>3.5	0.58	0.26–1.30	0.182	0.51	0.28–0.94	0.031
**CRP (mg/dL)**						
≤2	Ref			Ref		
>2	3.08	1.55–6.09	0.001	1.29	0.69–2.42	0.419
**D-dimer (μg/mL)**						
≤1	Ref			Ref		
>1	1.92	0.67–5.50	0.225	0.78	0.36–0.68	0.52

*Abbreviations:* SBP: systolic blood pressure; HR: heart rate; CGT: capillary glycemia test; CCI: age adjusted Charlson comorbidity index; Hb: hemoglobin; NLR: neutrophil/lymphocyte ratio; LDH: lactate dehydrogenase; CRP: C-reactive protein. CI: confidence interval; RR: relative risk.

### 3.5 Risk factors for in-hospital complications in the multivariate analysis

In the multivariate analysis for in-hospital complications, stroke severity retained the association but decreased in strength from 1.9 to 1.76. (RR = 1.76; 95% CI, 1.09–2.86; p = 0.022) and albumin levels >3.5 mg/dL which went from 0.45 to 0.53 (RR = 0.53; 95% CI, 0.36–0.79; p = 0.002) were maintained after adjusting for age, sex and stroke risk factors (Table 8).

**Table 8:** Multivariate analysis of risk factors for in-hospital complications in patients with acute ischemic stroke.

Variable	RR	95% CI	P value
**Sex**			
Female	Ref.		
Male	1.25	0.75–2.10	0.393
**Age**	1.02	0.99–1.04	0.086
**Hypertension**			
No	Ref.		
Yes	0.66	0.40–1.07	0.094
**Diabetes**			
No	Ref.		
Yes	1.06	0.65–1.72	0.82
**Stroke history**			
No	Ref.		
Yes	0.93	0.46–1.86	0.846
**HR (beat/min)**			
≤90	Ref.		
>90	1.41	0.88–2.27	0.153
**Stroke severity (NIHSS)**			
≤10	Ref.		
>10	1.76	1.09–2.86	0.022
**NLR**			
<3	Ref		
≥3	1.29	0.8–2.08	0.295
**Albumin (g/dL)**			
≤3.5	Ref		
>3.5	0.53	0.36–0.79	0.002

Multivariate analysis obtained with Poisson regression, parsimonic model. Abbreviations: NLR: neutrophil/lymphocyte ratio; CI: confidence interval; RR: relative risk.

In the exploratory analysis by type of complication, Hb (RR = 0.86; 95% CI, 0.73–0.99; p = 0.047) was a statistically significant protective factor for neurological complications, while in the analysis of infectious complications, all factors except for the history of prior medication for cardiovascular disease lost statistical significance (RR = 2.79; 95% CI, 1.16–6.71; p = 0.022), after adjusting for the confounders (Table 9).

**Table 9:** Multivariate analysis of risk factors for neurological and infectious complications in patients with acute ischemic stroke.

Variable	Neurological complications			Infectious complications		
	RR	95% CI	P value	RR	95% CI	P value
**Sex**						
Female	Ref.			Ref.		
Male	0.88	0.39–2.00	0.768	0.89	0.49–1.61	0.69
**Age**	0.99	0.95–1.03	0.655	1.01	0.99–1.04	0.33
**Hypertension**						
No	Ref.			Ref.		
Yes	0.88	0.40–1.98	0.772	0.97	0.52–1.83	0.931
**Diabetes**						
No	Ref.			Ref.		
Yes	0.76	0.35–1.67	0.502	0.95	0.50–1.82	0.877
**Stroke history**						
No	Ref.			Ref.		
Yes	1.14	0.49–2.64	0.763	0.67	0.25–1.75	0.411
**Previous medication**						
No				Ref.		
Yes	–			2.79	1.16–6.71	0.022
**Stroke severity (NIHSS)**						
≤10	Ref.					
>10	1.56	0.72–3.40	0.26	–		
**Hb (g/dL)**	0.86	0.73–0.99	0.047	–		
**CRP (mg/dL)**						
≤2	Ref					
>2	2.49	0.97–6.38	0.057	–		
**Albumin (g/dL)**						
≤3.5				Ref		
>3.5	–			0.67	0.35–1.28	0.225

*Abbreviations:* Hb: hemoglobin; CRP: C-reactive protein.

## 4 Discussion

The main objective of this study was to estimate the risk factors for in-hospital complications in patients with acute ischemic stroke hospitalized in a national reference hospital in Peru. As main findings, we found that approximately one in three patients presented in-hospital complications (32.3%) and the severity of the acute ischemic stroke defined by an NIHSS scale >10 was a risk factor and an albumin level >3.5 mg/dL was a protective factor for the development of in-hospital complications.

The frequency of in-hospital complications in the present study was lower than that reported by Pandian et al. in India (45.9%) and by Langhorne et al. in Scotland (85%), although these studies included patients with both ischemic and hemorrhagic stroke. They also considered pain and psychological complications [5,6]. Our study only included patients with acute ischemic stroke, as we consider that the clinical outcomes of patients with ischemic and hemorrhagic stroke could be heterogeneous [15].

In recent years, there has been a downward trend in the reporting of in-hospital complications, probably due to the optimization of stroke care in the different health systems. Although the highest mortality and disability rates are reported in Latin America and in many low- and middle-income countries [2], in recent years there have been joint efforts to improve this situation in this part of the world [16,17].

In the present study approximately 1 in 5 patients (22.4%) presented an infectious complication. This is consistent with what has been described in studies conducted in India, Scotland, and Argentina, in which infectious complications were the most frequent, with incidences of 34.4%, 65%, and 20%, respectively [5,6,9].

These findings are important, since most of the infectious complications are of the respiratory and urinary tract type, and these can be prevented with adequate education and training of the medical, nursing and technical personnel involved in the care of patients with stroke [18,19].

In the present study and others, the severity of stroke (NIHSS > 10) has shown to be an important risk factor [6,8,9]. This finding can be explained by the fact that the patients with greater stroke severity require different care and greater in-hospital monitoring which may lead to a higher risk of presenting complications during hospitalization. It is therefore important for patients admitted for stroke to be evaluated or admitted early to a stroke or neurological care unit if there is no physical space available in the ED [20,21]. In the case of our patients, the median stay in the ED was 2 days, which reflects the prompt referral to a neurological care unit.

Another finding in the present study was that albumin levels >3.5 mg/dL were found to be a protective factor for in-hospital complications. This marker was previously explored by Pandian et al. who found an association in the bivariate analysis but without statistical significance in the multivariate analysis [6]. The most plausible explanation is that serum albumin levels are a laboratory sign of the nutritional status of patients, and thus, patients with low albumin levels would have a higher risk of hospital complications and a worse prognosis as seen in similar pathologies [22,23].

In the exploratory analysis of the risk factors for the most frequent in-hospital complications (neurological and infectious), statistical significance was obtained for Hb as a protective factor for neurological complications, which, unlike previous studies in which the variable of anemia was considered, we believe Hb values should be analyzed as a numerical variable. Previous articles have shown that the presence of anemia increases the risk of mortality in patients with stroke and is a risk factor for in-hospital complications [6,24]. Although the mechanism for this has yet to be defined, it has been postulated that the decrease in oxygen supply in patients with anemia in a state of stress caused by the stroke, which requires an increase in oxygen levels, could predispose these patients to medical complications [6]. The history of prior medication for cardiovascular disease was a risk factor for the presence of infectious complications. This result can be explained in that the history of receiving previous medication is related to the severity of these comorbidities, which could condition the patients to presenting greater susceptibility to infectious processes, such as those reported in the present cohort.

Regarding clinical outcomes, the in-hospital mortality in the present study was 6.2%. This value is lower than that reported in previous articles in Peru [[25], [26], [27], [28]], such as the study carried out by Castañeda et al. in which a mortality of 13.6% was reported in patients with acute ischemic stroke admitted to a public hospital of national reference in the period 2000–2009. These findings are consistent with more recent data obtained in other Latin American countries which reported a decrease in hospital mortality due to stroke likely explained by the improvements in the care of these patients in recent years [29,30].

Our study also showed that almost half of the patients had a poor functional prognosis at discharge. However, the 5 patients who received intravenous reperfusion treatment in the acute stage achieved functional independence. This could be associated with a lower frequency of in-hospital complications; although this effect was not measured in our study, since it has already been shown that intravenous and mechanical reperfusion therapies are cost-effective strategies for reducing mortality, complications, and disability in patients with stroke [[31], [32], [33]].

This study has some limitations. Since the data were obtained from medical records, some laboratory variables were not available (CGT, LDH, D-dimer), and thus, certain variables could not be included in the multivariate regression models. The study was carried out with the main objective of estimating the risk factors for in-hospital complications, therefore the lack of statistical significance in the analyses of the most frequent types of complications may have been due to a purely statistical and not an epidemiological problem. Likewise, the follow-up period was limited to the time of hospitalization and did not allow analyses of association with the functional prognosis after hospital discharge.

Despite the above limitations, this study has some strengths. A significant number of patients was included because the study was performed in a national reference hospital. In addition, a complete analysis of different risk factors with global complications was carried out and factors were explored according to the most frequent types. Likewise, complete data of most of the variables were available due to adequate registration using the electronic medical record system.

## 5 Conclusions

In conclusion, this cohort study reports a high rate of in-hospital complications in patients presenting to the ED with stroke, among which infectious and neurological complications were of note as the most frequent. Stroke severity was a risk factor and albumin levels >3.5 mg/dL were a protective factor for the incidence of in-hospital complications in patients with acute ischemic stroke. Further studies are needed to confirm these results and develop stroke care systems that consider these markers to provide deferred care with stricter monitoring and prevention of hospital complications.

## Author contribution statement

Miguel A. Vences: Conceived and designed the experiments; Performed the experiments; Analyzed and interpreted the data; Contributed reagents, materials, analysis data; Wrote the paper.

Virgilio E. Failoc-Rojas: Performed the experiments; Analyzed and interpreted the data; Contributed reagents, materials, analysis data; Wrote the paper.

Diego Urrunaga-Pastor: Performed the experiments; Analyzed and interpreted the data; Wrote the paper.

Yamilée Hurtado-Roca: Conceived and designed the experiments; Performed the experiments; Contributed reagents, materials, analysis data; Wrote the paper.

## Data availability statement

Data will be made available on request.

## Declaration of interest’s statement

The authors declare that they have no known competing financial interests or personal relationships that could have appeared to influence the work reported in this paper.

## Additional information

No additional information is available for this paper.

## References

[1] Johnson C.O., Nguyen M., Roth G.A., Nichols E., Alam T., Abate D. (2019). Global, regional, and national burden of stroke, 1990–2016: a systematic analysis for the Global Burden of Disease Study 2016. Lancet Neurol..

[2] Martins S.C.O., Sacks C., Hacke W., Brainin M., Figueiredo F. de A., Pontes-Neto O.M. (2019). Priorities to reduce the burden of stroke in Latin American countries. Lancet Neurol..

[3] Feigin V.L., Brainin M., Norrving B., Martins S., Sacco R.L., Hacke W. (2022 Jan 1). World Stroke Organization (WSO): global stroke fact sheet 2022. Int. J. Stroke.

[4] Avezum Á., Costa-Filho F.F., Pieri A., Martins S.O., Marin-Neto J.A. (2015 Dec 1). Stroke in Latin America: burden of disease and opportunities for prevention. Global Heart.

[5] Langhorne P., Stott D.J., Robertson L., MacDonald J., Jones L., McAlpine C. (2000). Medical complications after stroke. Stroke.

[6] Pandian J.D., Kaur A., Jyotsna R., Sylaja P.N., Vijaya P., Padma M.V. (2012). Complications in acute stroke in India (CAST-I): a multicenter study. J. Stroke Cerebrovasc. Dis..

[7] Adeoye O., Nyström K.V., Yavagal D.R., Luciano J., Nogueira R.G., Zorowitz R.D. (2019). Recommendations for the establishment of stroke systems of care: a 2019 update. Stroke.

[8] Garavelli F., Ghelfi A.M., Kilstein J.G. (2021). Utilidad del score NIHSS como predictor de complicaciones intrahospitalarias no neurológicas en ictus isquémico. Med. Clínica.

[9] Guarnaschelli M., Lucero N., Andreata N.M., Buonanotte M.C., Atalah D., Deabato C. (2013). Factores de riesgo y complicaciones extraneurológicas de pacientes internados por Accidente Cerebrovascular en el Hospital Nacional de Clínicas de Córdoba. Rev. Fac. Cien. Med. Univ. Nac Cordoba.

[10] Elm E von, Altman D.G., Egger M., Pocock S.J., Gøtzsche P.C., Vandenbroucke J.P. (2007). The Strengthening the Reporting of Observational Studies in Epidemiology (STROBE) statement: guidelines for reporting observational studies. Lancet.

[11] Charlson M., Szatrowski T.P., Peterson J., Gold J. (1994). Validation of a combined comorbidity index. J. Clin. Epidemiol..

[12] Banks J.L., Marotta C.A. (2007). Outcomes validity and reliability of the modified Rankin scale: implications for stroke clinical trials. Stroke.

[13] Lyden P. (2017). Using the national Institutes of health stroke scale. Stroke.

[14] Adams H.P., Bendixen B.H., Kappelle L.J., Biller J., Love B.B., Gordon D.L., Marsh E.E. (1993). Classification of subtype of acute ischemic stroke. Definitions for use in a multicenter clinical trial. TOAST. Trial of Org 10172 in Acute Stroke Treatment. Stroke.

[15] Chiu D., Peterson L., Elkind M.S.V., Rosand J., Gerber L.M., Silverstein M.D. (2010). Comparison of outcomes after intracerebral hemorrhage and ischemic stroke. J. Stroke Cerebrovasc. Dis..

[16] Martins S.C.O., Lavados P., Secchi T.L., Brainin M., Ameriso S., Gongora-Rivera F. (2021). Fighting against stroke in Latin America: a joint effort of medical professional societies and governments. Front. Neurol..

[17] Ameriso S.F., Reynoso F. (2016). Aumentando la prevención y el tratamiento del accidente cerebrovascular en el continente americano: Declaración de Santiago de Chile, 31 de octubre del 2015. Neurol. Argentina.

[18] Kjörk E.K., Gunnel C., Lundgren-Nilsson Å., Sunnerhagen K.S. (2019). Experiences, needs, and preferences for follow-up after stroke perceived by people with stroke and healthcare professionals: a focus group study. PLOS ONE.

[19] Clare C.S. (2020). Role of the nurse in acute stroke care. Nurs. Stand..

[20] Collaboration S.U.T. (2013). https://www.cochranelibrary.com/cdsr/doi/10.1002/14651858.CD000197.pub3/full.

[21] Svendsen M.L., Ehlers L.H., Ingeman A., Johnsen S.P. (2012). Higher stroke unit volume associated with improved quality of early stroke care and reduced length of stay. Stroke.

[22] Manolis A.A., Manolis T.A., Melita H., Mikhailidis D.P., Manolis A.S. (2022). Low serum albumin: a neglected predictor in patients with cardiovascular disease. Eur. J. Intern. Med..

[23] Lyons O., Whelan B., Bennett K., O’Riordan D., Silke B. (2010). Serum albumin as an outcome predictor in hospital emergency medical admissions. Eur. J. Intern. Med..

[24] Li Z., Zhou T., Li Y., Chen P., Chen L. (2016). Anemia increases the mortality risk in patients with stroke: a meta-analysis of cohort studies. Sci. Rep..

[25] Málaga G., De La Cruz-Saldaña T., Busta-Flores P., Carbajal A., Santiago-Mariaca K. (2018). La enfermedad cerebrovascular en el Perú: estado actual y perspectivas de investigación clínica. Acta Méd. Peru..

[26] Castañeda-Guarderas A., Beltrán-Ale G., Casma-Bustamante R., Ruiz-Grosso P., Málaga G. (2011). Registry of patients with stroke stated in a public hospital of Peru, 2000-2009. Rev. Peru. Med. Exp. Salud Pública.

[27] Hernández-Vásquez A., Díaz-Seijas D., Espinoza-Alva D., Vilcarromero S. (2016). Análisis espacial de la Mortalidad distrital por enfermedades cardiovasculares en las provincias de Lima y Callao. Rev. Peru. Med. Exp. Salud Pública.

[28] Ministerio de Salud. Centro Nacional de Epidemiología, Prevención y Control de Enfermedades. Análisis de las causas de mortalidad en el Perú, 1986 - 2015 [Internet]. [cited 2023 Apr 4]. Available from: https://www.gob.pe/institucion/minsa/informes-publicaciones/279665-analisis-de-las-causas-de-mortalidad-en-el-peru-1986-2015.

[29] Soto Á., Guillén-Grima F., Morales G., Muñoz S., Aguinaga-Ontoso I., Vanegas J. (2022). Trends in mortality from stroke in Latin America and the Caribbean, 1979–2015. Global Heart.

[30] Lucci F.R., Lereis V.P., Ameriso S., Povedano G., Díaz M.F., Hlavnicka A. (2013). Mortalidad Intrahospitalaria por Accidente cerebrovascular. Medicina (Buenos Aires).

[31] de Souza A.C., Martins S.O., Polanczyk C.A., Araújo D.V., Etges A.P.B., Zanotto B.S. (2021). Cost-effectiveness of mechanical thrombectomy for acute ischemic stroke in Brazil: results from the RESILIENT trial. Int. J. Stroke.

[32] Lenz-Alcayaga R., Paredes-Fernández D., Hernández-Sánchez K., Valencia-Zapata J.E. (2021). Cost-utility analysis: mechanical thrombectomy plus thrombolysis in ischemic stroke due to large vessel occlusion in the public sector in Chile. Medwave.

[33] Turk A.S., Turner R., Spiotta A., Vargas J., Holmstedt C., Ozark S. (2015). Comparison of endovascular treatment approaches for acute ischemic stroke: cost effectiveness, technical success, and clinical outcomes. J. Neurointerventional Surg..

